# Help-Seeking Behaviors Among Older Adults: A Scoping Review

**DOI:** 10.1177/07334648211067710

**Published:** 2022-02-13

**Authors:** Kelly Teo, Ryan Churchill, Indira Riadi, Lucy Kervin, Andrew V. Wister, Theodore D. Cosco

**Affiliations:** 1 Department of Gerontology, 33507 Simon Fraser University, Vancouver, BC, Canada; 2 Gerontology Research Centre, 416185 Simon Fraser University, Vancouver, BC, Canada; 3 Oxford Institute of Population Ageing, 416185 University of Oxford, Oxford, UK

**Keywords:** health behaviors, health outcomes, diversity and ethnicity, help-seeking, scoping review

## Abstract

Although older adults may experience health challenges requiring increased care, they often do not ask for help. This scoping review explores the factors associated with the help-seeking behaviors of older adults, and briefly discusses how minority ethnic populations can face additional challenges in help-seeking, due to factors such as language barriers and differing health beliefs. Guided by Arksey and O’Malley’s scoping review framework and the Preferred Reporting Items for Systematic Reviews and Meta-AnalysesScoping Review guidelines, a systematic search of five databases was conducted. Using a qualitative meta-synthesis framework, emergent themes were identified. Data from 52 studies meeting inclusion criteria were organized into five themes: formal and informal supports, independence, symptom appraisal, accessibility and awareness, and language, alternative medicine and residency. Identifying how factors, including independence and symptom appraisal, relate to older adults’ help-seeking behaviors may provide insights into how this population can be supported to seek help more effectively.

## Introduction

Efforts to understand why many older adults do not seek help, even while experiencing grave symptoms, have highlighted the importance of understanding older adults’ help-seeking behaviors for their physical and mental health challenges (Woods et al., 2005). Older adults are less likely to access mental health services than their younger counterparts and even among those that do seek help, older adults are less likely to be offered treatment for their mental health challenges (Mackenzie et al., 2008; Woods et al., 2005). Such disparities in healthcare access have led to further research and efforts to support efficient and early help-seeking behavior among older adults, including the development of personal emergency response systems and the introduction of health checks in general practice (Porter & Markham, 2012; Woods et al., 2005). Even so, disparities in healthcare access and a lack of help-seeking continues to persist (Woods et al., 2005).

Failure to exhibit and appropriately identify help-seeking behavior delays opportunities to diagnose or treat older patients in a timely manner, which may further exacerbate symptoms and increase future care costs (Arthur-Holmes et al., 2020; Blakemore et al., 2018). This is especially a concern among minority ethnic older adults, whose continued underrepresentation in research leads to a poor understanding of what strategies may improve their help-seeking behavior and subsequent health outcomes (George et al., 2014; Korte et al., 2011). Compared to their counterparts, minority ethnic groups underutilize healthcare services, despite demonstrating a greater need for services (Greenwood & Smith, 2015; Walton & Anthony, 2017).

This scoping review aims to garner an in-depth understanding of the help-seeking behaviors of older adults, and to further explore those behaviors as they present in minority ethnic older adults. For this review, help-seeking behavior is defined as: any action taken by an older adult who perceives themselves as having a physical or mental health challenge, with the intent to find an appropriate remedy. The type of support that individuals pursue can include seeking formal support services (e.g., from clinicians, psychologists, and religious leaders) or informal support services (e.g., from family and friends). This review is meant to identify the ways in which older adults exhibit (or do not exhibit) help-seeking behaviors, and how they may experience barriers (and facilitators) from informal and formal supports.

## Methods

This scoping review adhered to the Preferred Reporting Items for Systematic Reviews and Meta-Analyses extension for Scoping Reviews guidelines (Tricco et al., 2018; Supplementary File 1) and is registered with the Open Science Framework (Teo, 2020). Further methodological details, additional definitions, and the published protocol for this scoping review are available online (Teo et al., 2021). The methods were also informed by Arksey and O’Malley (2005), which are described below.

### Identifying the Research Question

This review was undertaken with one main research question: Which factors are associated with help-seeking behavior among older adults? An additional sub-question was also included: How do cultural backgrounds, values, and differences influence help-seeking behavior among various older adult populations?

### Identifying Relevant Studies

The databases MEDLINE/PubMed, Web of Science, PsycINFO, CINAHL, and Scopus were searched from January 2005 to the date of search commencement in January 2021. Separate search strategies were used to address the two research questions, using three key concepts: “help-seeking behavior,” “older adults,” and “ethnic minorities.” Further details of the search strategy are detailed in Supplementary File 2. No language restrictions were imposed.

### Study Selection

After removing duplicates in EndNote, a pilot screen of 50 articles was conducted to ensure consistency and reliability. Two reviewers then conducted title/abstract and full-text screens of the same articles independently, based on the eligibility criteria (Table 1). Discrepancies between reviewers were reconciled through discussion and consultation with a third reviewer when necessary. Reference lists were hand-searched for potential missing articles.

**Table 1.** Inclusion and Exclusion Criteria.

Inclusion Criteria	Exclusion Criteria
• Full-text and peer-reviewed studies	• Systematic reviews, scoping reviews, opinion letters, conference proceedings, and dissertations
• Address the help-seeking behaviors of older adults	• Population dyads (e.g., includes both younger (<65) and older adults, or the perspectives of caregivers and healthcare providers)
• Published from January 2005 to the date of search commencement in January 2021	
• Participants aged 65 years old or older	• Non-community dwelling older adults
• Participants engaging in help-seeking behaviors or experiencing barriers to seeking help for their physical or mental health challenges	• Non-human studies

### Charting the Data

An Excel data extraction spreadsheet was created to extract the following data: authorship, year/journal of publication, population characteristics, location, methodology, limitations, help-seeking barriers or facilitators and any other pertinent information.

### Collating, Summarizing, and Reporting the Results

Following a qualitative meta-synthesis framework (Erwin et al., 2011), each study was carefully read and help-seeking factors were matched and compared with subsequent articles. They were then organized in the following manner: first-order constructs were recorded as direct factors, quotes, and findings from the studies themselves, second-order constructs were interpretive themes that formed the basis of each category, and third-order constructs were developed from the aggregation of multiple categories that led to the development of new themes.

## Results

### Included Studies

**Figure 1.**
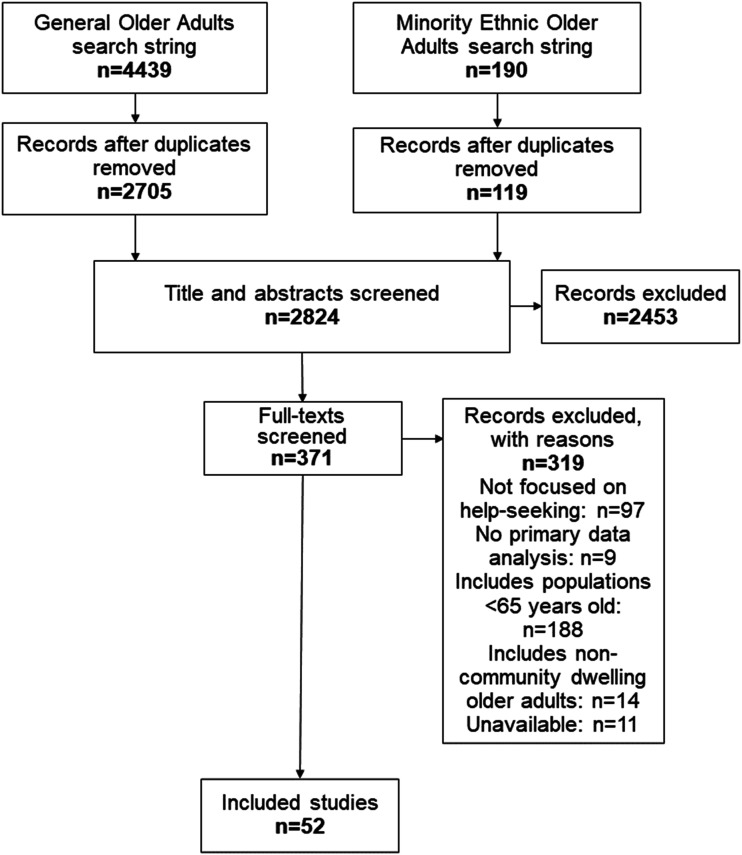
Preferred reporting items for systematic reviews and meta-analyses flowchart.

From the searches, 2824 unique articles were identified. A total of 52 articles met inclusion criteria (Figure 1).

### Study Characteristics

The number of studies was highest in Europe (*n* = 18), followed by North America (*n* = 16), Asia (*n* = 12), Australia (*n* = 5), and New Zealand (*n* = 1). Most studies used qualitative methods (*n* = 26; Table 2), followed by quantitative (*n* = 24; Table 3) and mixed methods (*n* = 2; Table 4). More papers focused on physical health challenges (e.g., chronic conditions, pain; *n* = 28), over mental health challenges (e.g., depression; *n* = 11). Thirteen studies discussed both physical and mental health challenges or discussed general health challenges. Tables 2–4 summarize the characteristics of included studies while key findings from each article regarding health challenges, help-seeking behaviors, barriers, and facilitators are summarized in Supplemental Table 1.

**Table 2.** Study Demographics: Qualitative Study Demographics.

Author(s)	Country	Study Population	N	Age
Altizer et al., 2014	United States	African American and White older adults in south-central North Carolina counties	62	≥65
Aubut et al., 2021	Canada	French-speaking Quebecers receiving treatment at a public addiction center	11	≥65
Begum et al., 2012	United Kingdom	Residents of southeast London	18	≥65
Canvin et al., 2018	United Kingdom	Older adults living in North West England and North Wales	40	68–95
Chen, 2020	Taiwan	Older adults recruited from community day care centers, living in rural areas of Taiwan	35	≥65
Chung et al., 2018	United States	Older Korean immigrants living in the Seattle metropolitan area	17	67–79
Clarke et al., 2014	United Kingdom	Older adults in North-East Scotland	23	66–89
Dollard et al., 2014	Australia	Older women living in Adelaide, Australia	11	65–87
Elias and Lowton 2014	London	Older adults recruited from 2 -day centers in South-East London	15	80–93
Frost et al., 2020	United Kingdom	Frail older people recruited from general practices in the United Kingdom	28	75–88
Gore-Gorszewska, 2020	Poland	Polish residents	30	65–82
Hannaford et al., 2019	Australia	Older adults from New South Wales, Australia	14	65–89
Hurst et al., 2013	United Kingdom	Older people recruited from day centers, advertisements, or snowball recruitment	9	66–85
Johnston et al., 2010	Australia	Volunteers who had sustained a fall in the previous 6 months	31	≥65
Kelly et al., 2011	United States	Caucasian older adults living in the metropolitan areas of Phoenix and San Francisco	93	65–80
Kharicha et al., 2013	London	Patients from a general practice located in suburban London	76	≥65
Lawrence et al., 2006	United Kingdom	Black Caribbean, South Asian, and white British older adults	110	≥65
Lee et al. 2020	Singapore	Older adults receiving public financial assistance and living in the Chin Swee residential estate	11	66–88
Makris et al., 2015	United States	Racially diverse population of older adults living in Connecticut or New York City	93	≥65

Author(s)	Country	Study Population	N	Age
Miller et al., 2016	Canada	Canadian seniors who visited an Emergency Department in Ontario, because of a fall, but were not hospitalized	20	65–88
Mukherjee, 2019	India	Migrant Bangladeshi elderly women living in North 24 Parganas, West Bengal, India	104	≥65
Polacsek et al., 2019	Australia	Older people receiving treatment and/or support for a formal diagnosis of moderate depression	32	65–82
Schaller et al., 2020	Norway	Native Norwegian adults of all sexual orientations, with or without partners	32	65–85
Stoller et al., 2011	United States	African American and White adults from 3 rural counties in south-central North Carolina	62	65–92
Tsai and Tsai 2007	Taiwan	Elders living alone in a remote area of eastern Taiwan	9	65–90
Waterworth et al., 2018	New Zealand	Older adults with multimorbidities, recruited from general practices in New Zealand	21	66–90

**Table 3.** Study Demographics: Quantitative Study Demographics.

Author(s)	Country	Study Population	N	Age
Bonnewyn et al., 2009	Europe	Older adults living in Belgium, France, Germany, Italy, The Netherlands, and Spain	4401	≥65
Djukanović et al., 2015	Sweden	Residents of Sweden	6659	65–80
Eriksson-Backa et al., 2018	Finland	Finnish seniors	273	65–79
Garg et al., 2017	United States	Medicare beneficiaries	11,270	≥65
Garrido et al., 2011	United States	Older community-dwelling adults in the United States	1681	≥65
Gur-Yaish et al., 2016	Israel	Older Jewish population in Israel	509	66–92
Hartvigsen et al., 2006	Denmark	Danish twins	1844	72–102
Hohls et al., 2021	Germany	Participants of the AgeQualiDe-Study (a German study on the oldest-old primary care patients)	155	≥85
Horng et al., 2014	Taiwan	Older Taiwanese people	2715	≥65
Kagan et al., 2018	Israel	Older men in Israel who speak Hebrew	256	≥65
Krishnan and Lim 2012	Singapore	Single elderly Indian men who speak Tamil and receive state financial assistance	75	65–86
Lau et al., 2014	China	Chinese older adults recruited from health centers in Macao, China	301	65–91

Author(s)	Country	Study Population	N	Age
Lee et al. 2005	Korea	Elderly males without prior prostate surgery, prostate cancer, or bladder disease	417	65–84
Lee et al. 2012	United States	Older adults who have health insurance and a usual source of care	85,015	≥65
Makam et al., 2016	United States	Patients hospitalized at medical centers in central Massachusetts	1542	≥65
McCabe et al., 2017	United States	Patients living in the Midwestern United States, recruited from a primary care clinic	180	65–91
McGowan and Midlarsky 2012	United States	White and Black older adults of various religious affiliations	307	65–94
Mechakra-Tahiri et al., 2011	Canada	French-speaking older adults living in Quebec	2811	≥65
Murata et al., 2010	Japan	Older adults who were literate and understood Japanese, living in 6 municipalities from 3 prefectures	15,302	65–100
Pickard and Guo 2008	United States	Participants living in a naturally occurring retirement community (NORC) in Missouri	317	65–95
Pickard and Tang 2009	United States	Participants living in a Naturally occurring Retirement community (NORC) in Missouri	317	65–95
Schneider et al., 2014	Australia	Clients attending 3 low-vision clinics in Sydney, Australia	169	≥65
Stenzelius et al., 2006	Sweden	Older adults who reported having difficulties controlling urine and/or other urinary symptoms	771	≥75
Stenzelius et al., 2007	Sweden	Older adults who reported having difficulties controlling feces	248	75–96

**Table 4.** Study Demographics: Mixed Methods Study Demographics.

Author(s)	Country	Study Population	N	Age
Horton and Dickinson 2011	England	Chinese older people based in central London, England	30	≥65
Hwang and Jeong 2012	South Korea	First-time AMI patients recruited from the cardiovascular unit of a national university hospital	165	65–89

## Overview of Findings

### Formal and Informal Supports

From the themes that emerged from this review, distinctions between seeking help from informal and formal supports are important to discuss. Within formal support, several articles described how mistrust, perceptions of a physician’s role, and past interactions with formal healthcare providers can negatively influence older adults’ help-seeking behaviors. Here, the notion that physicians could either not help at all or any more than they already were helping was prevalent (Dollard et al., 2014; Elias & Lowton, 2014; Garg et al., 2017). This perspective was reinforced by past experiences of help-seeking, when misdiagnosis or non-diagnosis occurred (Clarke et al., 2014; Polacsek et al., 2019). Furthermore, dismissal of health concerns and a lack of respect exhibited by a healthcare provider reinforced older adults’ reluctance to share any new challenges at subsequent consultations (Gore-Gorszewska, 2020; Lawrence et al., 2006; Makris et al., 2015). Conversely, a positive relationship with healthcare providers was a facilitator to help-seeking. For example, positive views of both the healthcare system and psychological treatment were found to increase the likelihood of using such services (Begum et al., 2012; Gur-Yaish et al., 2016). Feeling heard by the providers, gaining a sense of security, and being given practical information and assistance were deemed to be important components of this relationship (Clarke et al., 2014; Waterworth et al., 2018), as was older adults being included in the decision-making process (Polacsek et al., 2019).

When it came to informal supports, the availability of such often reduced the need or desire for formal support, as the older adult’s concerns were alleviated by those around them (Begum et al., 2012; Hohls et al., 2021; Lau et al., 2014; Mechakra-Tahiri et al., 2011). Here, social support allowed opportunities to exchange health information, disclose, and receive endorsements/recommendations for treatments (Aubut et al., 2021; Begum et al., 2012; Chen, 2020; Chung et al., 2018; Frost et al., 2020; Hurst et al., 2013; Lawrence et al., 2006; McCabe et al., 2017; Mechakra-Tahiri et al., 2011; Polacsek et al., 2019; Schneider et al., 2014). Unsolicited social support also facilitated help-seeking, when decisions for the older adult were made on their behalf by others (Begum et al., 2012; Canvin et al., 2018; Lawrence et al., 2006), simply offered or given (Miller et al., 2016) or when family and friends would influence, persuade, or coerce older adults to seek help (Dollard et al., 2014; Elias & Lowton, 2014; Hurst et al., 2013; Johnston et al., 2010).

### Independence

Older adults often indicated the preference to not want others involved in their health challenges when help-seeking was viewed as a threat to their independence (Aubut et al., 2021; Canvin et al., 2018; Elias & Lowton, 2014; Frost et al., 2020; Johnston et al., 2010; Lee et al., 2020; Miller et al., 2016). Related to this were desires to be seen in a positive light and notions of not wanting to bother others or be a burden (Begum et al., 2012; Canvin et al., 2018; Chen, 2020; Clarke et al., 2014; Dollard et al., 2014; Elias & Lowton, 2014; Gore-Gorszewska, 2020; Horton & Dickinson, 2011; Hurst et al., 2013; Johnston et al., 2010; Lawrence et al., 2006; Lee et al., 2020; Miller et al., 2016). As such, for help-seeking to occur, older adults had to view this behavior as a functional way of increasing their own independence. For example, Johnston et al. (2010) reasoned that effective alarm users were more likely to be positive about using such aids after a fall, as it improved an older adult’s confidence in living alone and provided reassurance to their families. Talking therapies and loved ones setting a routine with the older adult were also effective options that allowed participants to reach their own solutions and receive informal help without feeling like they were imposing on another’s schedule (Frost et al., 2020; Miller et al., 2016).

### Symptom Appraisal

When confronted with a symptom, older adults would commonly self-assess their health first to determine whether seeking help was necessary (Hurst et al., 2013; Hwang & Jeong, 2012; Tsai & Tsai, 2007; Waterworth et al., 2018). As such, there was often a delay in help-seeking as many underestimated the seriousness of their conditions (Garg et al., 2017; Murata et al., 2010) and would subsequently wait to see if the problems went away or attempt to self-manage their conditions, with informal support being a common first choice of help (Altizer et al., 2014; Begum et al., 2012; Canvin et al., 2018; Djukanović et al., 2015; Frost et al., 2020; Garrido et al., 2011; Hurst et al., 2013; Hwang & Jeong, 2012; Johnston et al., 2010; Lee et al., 2020; McCabe et al., 2017; Tsai & Tsai, 2007). For example, Kelly et al. (2011) found that despite acknowledgment of hearing impairment, those in the non-consulting group did not experience communication breakdowns or experienced them in predictable ways, resulting in lower levels of anxiety and a lack of help-seeking. Furthermore, experiencing comorbidities made issues such as activity-restricting back pain or sexual problems seem less of a health priority (Makris et al., 2015; Schaller et al., 2020; Schneider et al., 2014). During this symptom appraisal process, older adults would also often attribute their poor health as normal or inevitable due to age (Canvin et al., 2018; Clarke et al., 2014; Elias & Lowton, 2014; Frost et al., 2020; Gore-Gorszewska, 2020; Hurst et al., 2013; Hwang & Jeong, 2012; Kharicha et al., 2013; Lee et al., 2005; Makris et al., 2015; Mukherjee, 2019; Polacsek et al., 2019; Waterworth et al., 2018). This “demedicalization of health problems” (Elias & Lowton, 2014, p. 977–978) was further exacerbated and reinforced when older adults did present these issues to formal health professionals, only to be met with dismissal, limited treatment options, or ageist and patronizing comments (Frost et al., 2020; Gore-Gorszewska, 2020; Makris et al., 2015; Polacsek et al., 2019).

On the contrary, the decision to seek help from health professionals was based on several determinants, such as when symptoms were more noticeable/unfamiliar or when symptoms affected their daily lives (Altizer et al., 2014; Begum et al., 2012; Canvin et al., 2018; Clarke et al., 2014; Dollard et al., 2014; Elias & Lowton, 2014; Frost et al., 2020; Horng et al., 2014; Hurst et al., 2013; Hwang & Jeong, 2012; Kharicha et al., 2013; Lee et al., 2020; Makam et al., 2016; Mukherjee, 2019; Schneider et al., 2014; Stenzelius et al., 2006, 2007; Stoller et al., 2011; Waterworth et al., 2018). For example, one study found that those with painful physical symptoms were more likely to seek formal help (e.g., from mental health services) than those without (Bonnewyn et al., 2009), while another found that the duration and intensity of pain were associated with seeking help (Hartvigsen et al., 2006).

### Accessibility and Awareness

In regards to accessibility and awareness of formal services, older adults expressed related issues such as costs, long wait times, short consultation times or having busy schedules as help-seeking barriers (Djukanović et al., 2015; Dollard et al., 2014; Frost et al., 2020; Garg et al., 2017; Garrido et al., 2011; Hannaford et al., 2019; Kagan et al., 2018; Lawrence et al., 2006; Lee et al., 2012, 2020; Murata et al., 2010; Polacsek et al., 2019; Schneider et al., 2014; Waterworth et al., 2018). Location and lack of available transportation also made it more difficult for older adults to seek help, especially among those living in rural areas or those with physical constraints (Chen, 2020; Chung et al., 2018; Frost et al., 2020; Garg et al., 2017; Garrido et al., 2011; Gur-Yaish et al., 2016; Mukherjee, 2019; Murata et al., 2010; Polacsek et al., 2019; Waterworth et al., 2018). Furthermore, a lack of available information and awareness of services was evident (Aubut et al., 2021; Chung et al., 2018; Djukanović et al., 2015; Garrido et al., 2011; Gore-Gorszewska, 2020; Horton & Dickinson, 2011; Lee et al., 2020; Mukherjee, 2019; Polacsek et al., 2019; Tsai & Tsai, 2007), where limited knowledge also limited older adults’ understanding of the severity of their issue. This was particularly true for symptoms related to heart issues (Hwang & Jeong, 2012; McCabe et al., 2017).

To facilitate resource use and seeking activity, a greater understanding of resources/services, convenience of service use, and higher health literacy were notable factors (Eriksson-Backa et al., 2018; Polacsek et al., 2019). Issues of location and transportation led to the importance of being able to speak to someone on the phone for health-related supports, especially for those living in rural areas (Waterworth et al., 2018). However, the adoption of telephone consultations varied, with some experiencing challenges due to hearing problems or invoking fears of not knowing who they were speaking to (Frost et al., 2020).

### Language, Alternative Medicine, and Residency

Among minority ethnic populations, several factors, including language differences, were a notable impediment to help-seeking (Chung et al., 2018; Horton & Dickinson, 2011; Krishnan & Lim, 2012; Mukherjee, 2019; Tsai & Tsai, 2007). As Chung et al. (2018) describes, a lack of English proficiency and lack of information provided in Korean limited older Korean immigrants’ social activities, enhanced their preference for a Korean-speaking doctor, and increased their reliance on others for support. Similarly, Krishnan and Lim’s (2012) study on older Indian men living in Singapore found that those who experienced language discordance were more likely to be dissatisfied with care. Others expressed the notion that it would be inappropriate to share personal problems with strangers (Lau et al., 2014; Lawrence et al., 2006). Additional cultural factors and beliefs included the use of alternative medicine due to mistrust of American providers and Western medicines (Chung et al., 2018) and the idea that help-seeking was futile for reasons such as luck, sin, karma, or destiny (Horton & Dickinson, 2011; McGowan & Midlarsky, 2012; Mukherjee, 2019; Tsai & Tsai, 2007). However, this was not only unique to articles studying minority ethnic groups, as several others highlighted similar beliefs related to superstition/fatalism and the seeking of help from religious leaders over other formal sources (Hannaford et al., 2019; Hurst et al., 2013; Johnston et al., 2010; Lawrence et al., 2006; McGowan & Midlarsky, 2012; Pickard & Guo, 2008; Pickard & Tang, 2009).

Facilitators to help-seeking among minority ethnic older adults included a longer length of residency in the country where one immigrated, which led to an improved ability to communicate with healthcare providers in English and a greater familiarity with how to navigate the healthcare system (Chung et al., 2018). In addition, the availability of community organizations that offered translation services or home care helpers enhanced opportunities to access healthcare and programs, find information, and socialize with others (Chung et al., 2018).

## Discussion

This scoping review demonstrates several thematic areas that explicate why older adults avoid help-seeking when health challenges arise. Many factors overlap, highlighting the way in which a decision to seek help among older adults can be intricate and complex. For instance, once a decision to seek help has been made, informal supports are common first choices for help, which is consistent with Cantor’s (1979) hierarchical-compensatory model of social supports. In circumstances where informal supports are available, help-seeking can end when needs are adequately met by these individuals. Informal support may also encourage seeking of formalized support such as that of healthcare professionals, or a blending of informal and formal support seeking may occur. However, even if older adults are at a stage where they are willing to seek help from formal avenues, there are structural barriers (e.g., costs or location) that can prevent them from doing so (Chen, 2020; Chung et al., 2018; Johnston et al., 2010; Murata et al., 2010).

Further barriers identified by this review include negative perceptions of and relationships with healthcare providers and perceived threats to independence. This is consistent with reviews studying the help-seeking behaviors of other sub-populations, such as Indigenous communities (Fiolet et al., 2021), women with urinary incontinence (Koch, 2006), and men (Yousaf et al., 2015), where avoiding help is linked to barriers such as inappropriate responses from service providers, the need for independence and control, and fear related to shame and repercussions. Symptom appraisal was also a prevalent factor among the older adults in this review, with serious or novel symptoms prompting help-seeking. This finding is similar to the aforementioned reviews, with other groups also highlighting seriousness and the feeling like a crisis had been reached as a turning point in the decision to seek help (Fiolet et al., 2021; Koch, 2006).

A sub-focus on minority ethnic older adults highlights additional barriers that further inhibit this group’s ability to adequately seek help for their needs. These issues include cultural beliefs, immigration, and language, which are intermixed with issues of mistrust, structural barriers, and symptom appraisal. This pattern of help-seeking among older adults is similar to findings among younger minority ethnic populations, where traditional/cultural beliefs, and the employing of alternative strategies is often invoked (Richards, 2019; Rüdell et al., 2008). To our knowledge, this is the first scoping review conducted on this topic, and thereby provides a comprehensive overview of existing literature and the most prevalent help-seeking factors for older adults. In doing so, these findings highlight the importance of including minority ethnic populations in research and imply the need for strategies that reduce barriers to help-seeking at all levels: among older adults themselves, their social networks, and formal services.

### Limitations

Although the review findings will inform future research, our conclusions are limited by the methodological quality of the included studies and aspects of the review strategy itself. It is possible that there are studies that should have been captured in this search but were not, due to the search strategy, the lack of standardization of help-seeking terms or the indirect ways that help-seeking may have been addressed. Furthermore, 11 studies were unavailable to be retrieved through the institutional library accessible to the reviewers and requests sent to the authors for full-texts (via ResearchGate) were not answered, preventing possible inclusion of these studies and their results. The findings could have also been limited by the inclusion criteria, whereby articles excluding perspectives of stakeholders (e.g., caregivers) could have prevented a discussion of additional help-seeking factors. In addition, despite a secondary focus to explore the help-seeking behaviors of minority ethnic older adults, only nine articles were uncovered. The age cut-off of including only older adults aged 65 years and older may have introduced a selection bias that reduces the relevancy of these findings in countries with lower life expectancies, and thus should be a future research consideration. Furthermore, due to the ways in which language is constantly evolving, the search terms may have prevented the capturing of articles that identified specific populations or used terms such as people of color, Black and Minority Ethnic, or racially minoritized.

### Future Directions

In consideration of the knowledge gaps in this field, a greater focus on specific minority ethnic populations, help-seeking interventions, and evaluations of such interventions are needed. Greater education efforts among older adults and their networks are also necessary, in recognition of how older adults may inadequately appraise their health or be unaware of available services. Patient engagement strategies that allow older adults to remain as independent as possible can also prevent fears of disempowerment or a lack of control in decisions that occur because of help-seeking. Furthermore, greater training for providers serving the older adult population is needed, where respect for autonomy and diversity are salient considerations.

## Conclusion

Evidently, the help-seeking behaviors of older adults are complex interactions of various factors, considerations, and experiences. This scoping review has indicated a need to address the barriers that older adults experience when a need for help arises. It also suggests the need for comprehensive changes that involve the older adult themselves in decision-making when possible, while considering their relationships, cultural values, and beliefs in a holistic way. Through addressing and recognizing these factors, older adults can be better empowered and prepared to seek help, without delay.

## Supplemental Material

sj-pdf-1-jag-10.1177_07334648211067710 - Supplemental Material for Help-Seeking Behaviors Among Older Adults: A Scoping Review Click here for additional data file. Supplemental Material, sj-pdf-1-jag-10.1177_07334648211067710 for Help-Seeking Behaviors Among Older Adults: A Scoping Review by Kelly Teo, Ryan Churchill, Indira Riadi, Lucy Kervin, Andrew V. Wister, and Theodore D. Cosco in Journal of Applied Gerontology

sj-pdf-2-jag-10.1177_07334648211067710 - Supplemental Material for Help-Seeking Behaviors Among Older Adults: A Scoping Review Click here for additional data file. Supplemental Material, sj-pdf-2-jag-10.1177_07334648211067710 for Help-Seeking Behaviors Among Older Adults: A Scoping Review by Kelly Teo, Ryan Churchill, Indira Riadi, Lucy Kervin, Andrew V. Wister, and Theodore D. Cosco in Journal of Applied Gerontology

sj-pdf-3-jag-10.1177_07334648211067710 - Supplemental Material for Help-Seeking Behaviors Among Older Adults: A Scoping Review Click here for additional data file. Supplemental Material, sj-pdf-3-jag-10.1177_07334648211067710 for Help-Seeking Behaviors Among Older Adults: A Scoping Review by Kelly Teo, Ryan Churchill, Indira Riadi, Lucy Kervin, Andrew V. Wister, and Theodore D. Cosco in Journal of Applied Gerontology
