# Supplementary material for: Help-Seeking Behaviors Among Older Adults: A Scoping Review
Source: J Appl Gerontol. 2022 Feb 13;41(5):1500–10. doi: 10.1177/07334648211067710 (PMC9024019; doi:10.1177/07334648211067710)
Supplement: sj-pdf-1-jag-10.1177_07334648211067710 - Supplemental Material for Help-Seeking Behaviors Among Older Adults: A Scoping Review [file sj-pdf-1-jag-10.1177_07334648211067710.pdf]

Supplemental Table 1. Help-Seeking Characteristics & Factors among Older Adults

Author(s)	Type of help-seeking behavior(s)	Health challenge(s)	Help-seeking Barriers	Help-seeking Facilitators
Altizer et al. 2014	Health information seeking (e.g. from friends, healthcare providers & media sources)	General health challenges	-	Being confronted with unfamiliar/novel sumptoms or when a known treatment was not effective
Aubut et al. 2021	Asking for help from an addiction service centre	Substance use	Lack of knowledge about services and availability of services	Deteriorating health, desire to fill grandparent role, involvement of social supports, having a family doctor, available resources (e.g. addiction services)
Begum et al. 2013	Seeking help from a local specialist memory assessment service	Memory complaints	Viewing issues as not concerning, having causal beliefs that led to beliefs in lack of control, negative perceptions of general practitioners, stigma, pride, preference to cope alone or to use self-help strategies	Positive views of health system, having alternative pathways to care (e.g. medical surveillance through regular visits with general practitioner), available social supports, presence of comorbidities
Bonnewyn et al. 2009	Seeking mental health services, antidepressants, and benzodiazepines	Depression and chronic pain	-	Presence of major depressive episode or painful physical symptoms
Canvin et al. 2018	Various (e.g. medical and family-provided support, both paid or unpaid)	General health challenges	No perception of need, viewing decline as an inevitable consequence of age, viewing assistance as a threat to independence and inability to cope, not wanting to be a burden, passivity, lack of awareness	Reaching a point where deterioration could not be denied, impact of decline on usual activities and independence
Chen 2020	Medical treatment-seeking	Chronic conditions (e.g. diabetes, hypertension, arthritis, heart disease)	Low literacy, low awareness of illness and severity, cost, not wanting to be a bother to family, poor family living conditions, resignation to fate/folk remedies, poor doctor-patient communication	Availability of others for help and advice (e.g. family, neighbours, volunteers), having treatments close to home, increased seriousness of disease
Chung et al. 2018	Various (e.g. Health information seeking, complementary/alternative therapies, regular clinic visits)	Various (e.g. Chronic conditions, memory issues, mobility limitations)	Seeing oneself as healthy, mistrust, adapting to aging body, shorter immigration period, language barriers, lack of support and available information, cost, limited health insurance	Longer length of residency, community resources and organizations, social engagement, physicians motivating concerns about future falls and frailty, home care workers serving as source of health information, health coverage
Clarke et al. 2014	Seeking help from health professionals	Musculoskeletal chronic pain	Not wanting to bother or waste physician's time, the need to be really ill first, not wanting to be viewed as a 'complainer' or a 'fraud', issues associated with age, not getting a diagnosis for pain, assuming providers's dismissiveness	When pain becomes persistent/noticeable/repetitive, feeling listened to and heard
Djukanović et al. 2015	Seeking formal care (from a physician, district nurse, welfare officer, psychologist and physiotherapist)	Depression	Negative experiences from previous provider visits, difficulty getting through on the phone, symptoms got better on its own, cost, lack of awareness, time (e.g. had no time or waiting time too long)	-

Supplemental Table 1. Help-Seeking Characteristics & Factors among Older Adults

Author(s)	Type of help-seeking behavior(s)	Health challenge(s)	Help-seeking Barriers	Help-seeking Facilitators
Dollard et al. 2014	Seeking help from a general practitioner	Falls	Perceiving fall or fall-related injury as not serious, beliefs that general practitioner could not help, not wanting to "make a fuss", attribution of falls to extrinsic factors (e.g. uneven footpath, hurrying), discounting intrinsic risk factors (e.g. leg weakness, poor balance), not wanting to waste practitioners' time, untimely access to practitioner	Perceiving fall-related injury as serious (e.g. visible injuries or pain impairing mobility), persuasion/negotiation/coercion by others
Elias and Lowton 2014	Seeking medical care (e.g. from a general practitioner, at a hospital)	Various (e.g. severe pain, coughs)	Belief that doctors could not help, attributing problems to old age, desire not to burden others, valuing functional independence, negative accounts of receiving help when they did not seek it or being sent home despite seeking help at the hospital	Greatly impaired function, believing they were more vulnerable due to age, perceiving that physical access to the doctor was more difficult, being obliged by relatives
Eriksson-Backa et al. 2018	Health information seeking	General health challenges	-	Higher health information literacy, being female, higher education level
Frost et al. 2020	Various (e.g. from general practitioners, social workers, mental health professionals, adult children, support groups)	Frailty, depression and anxiety	Low expectations, normalizing decline and the aging experience, not experiencing improvement after seeking treatment, family conflict, finances, lack of awareness of available supports, threats to independence, fear of dependency on antidepressants, experiencing difficulties with telephone appointments or technology, being unable to access appointments, difficulty leaving homes	Moderate-to-severe symptoms, endorsement of treatments by others, face-to-face contact, delivery of services provided at home, locally or with transport provided
Garg et al. 2017	Seeing a doctor or other medical person	Various (e.g. chronic conditions, multimorbidity)	Low perceived severity of illness, costs, belief that doctors could not help, schedule or personal conflicts, trouble finding or getting to a doctor, high worry and fear of knowing the illness, and non-acceptance of insurance by the doctor	Higher age, non-smoking status, higher income, having a usual source of care, lower worry about personal health
Garrido et al. 2011	Seeking mental health care (e.g. from a physician, nurse, occupational therapist, psychiatrist, psychologist, social worker)	Various (e.g. mental illness, alcohol abuse, chronic conditions)	Problems went away by itself, desire to handle issues on their own, cost, low perceived seriousness, thinking treatment would not be useful, not knowing where to go for care, scheduling difficulties, limited insurance coverage, stigma	Perceiving a need for care, having a history of more chronic physical conditions, history of alcohol abuse or dependence, having private insurance
Gore-Gorszewska 2020	Seeking medical or psychological help	Sexual problems	Not recognizing symptoms or treating symptoms as part of aging, fear of doctors' disapproval or having problems dismissed, stigma, sexual problems considered less important than other medical conditions, lack of knowledge and awareness on how to access appropriate services	-

Supplemental Table 1. Help-Seeking Characteristics & Factors among Older Adults

Author(s)	Type of help-seeking behavior(s)	Health challenge(s)	Help-seeking Barriers	Help-seeking Facilitators
Gur-Yaish et al. 2016	Using psychotherapy	Depressive symptoms	-	Perceived need, positive attitudes toward psychotherapy use, accessibility to services, recommendations from primary physicians
Hannaford et al. 2019	Seeking psychological services	Mental health challenges	Reluctance to disclose mental health concerns, short consultation times, passivity, a focus on physical symptoms, lack of enquiry by the GP, low mental health literacy, lack of familiarity with psychotherapy, feeling that challenges should be dealt with individually, stigma	Experiencing trust and the absence of judgement, practitioner's providing advice/referral/initiating discussion of mental health problems, favorable help-seeking experiences in the past, access to social and professional networks linked with religious communities
Hartvigsen et al. 2006	Seeking medical care (e.g. from a general medical practitioner, medical specialist, hospital, chiropractor, physical therapist)	Neck and back pain	-	Higher duration and intensity of pain
Hohls et al. 2021	Receiving informal and/or formal support (e.g. from friends, relatives, or local services)	Psychological distress	-	Experiencing psychological distress or increased anxiety symptoms
Horng et al. 2014	Seeking medical attention	Fecal incontinence	-	Living in urban areas, mucous stool incontinence, frequent symptoms impacting social life/work life, anxiety, family interactions
Horton and Dickinson 2011	Various (e.g. using formal health services, telling adult children)	Falls	Embarassment, wanting to speak on positive events with friends, not wanting to worry adult children, belief in metaphysical explanations such as fatalism/luck/lay beliefs, attribution of illness with age, self-judgement, lack of information/knowledge about services, language & literacy barriers	Subsidized interventions, information presented via visual media (e.g. via television/DVD in Chinese, word of mouth), culturally appropriate interventions
Hurst et al. 2013	Health information-seeking (e.g. from newspapers, doctors, nurses, pharmacists, family, friends, internet)	General health challenges	Values and beliefs related to family history and superstition, perceiving the event as insignificant, viewing their condition as part of a normal aging process, fear, not wanting to bother the doctor	Change in health, the need for information around an actual or suspected diagnosis (self-assessment of symptoms usually done first), reading articles in the newspaper and having conversations with family/friends/health professionals, viewing event as serious, rising health concerns, past experiences and knowledge
Hwang and Jeong 2012	Going to the hospital	Acute myocardial infarction (AMI)	Lower education, non-ST-elevation MI, presence of preinfarction angina pain, attribution of symptoms to a non-cardiac origin, perceiving no heart attack threat, lack of knowledge/recognition of urgency of symptoms and risk, optimistic self-appraisal of symptoms, overconfidence about health, not wanting to bother or burden others	-

Supplemental Table 1. Help-Seeking Characteristics & Factors among Older Adults

Author(s)	Type of help-seeking behavior(s)	Health challenge(s)	Help-seeking Barriers	Help-seeking Facilitators
Johnston et al. 2010	Using a personal alarm to receive assistance	Falls	Performance of alarms not well understood, alarms viewed as obtrusive/uncomfortable/unnecessary, expressions of fatalism/resignation/denial of fall risk, cost, negative feelings of being a burden, threats to independence and identity, fear of going to the hospital and not being able to return home	Perceived benefits of personal alarms (e.g. personal safety, improved independence and reassurance), alarm use motivated by previously bad experiences (e.g. previous injury), positive experience of receiving quick medical attention after using the alarm, influence of family/health professionals
Kagan et al. 2018	Seeking help from a social worker	General health challenges	Stigma, higher education, lower self-rated health	Self-reported loneliness, better self-rated health, positive attitudes towards social workers, prior experience with receiving professional help
Kelly et al. 2011	Consulting hearing services	Hearing impairments	Not experiencing communication breakdowns or experiencing them in predictable ways (resulting in low cognitive anxiety and lack of consulting)	Being unable to make useful predictions about communication events, provision of hearing aids that reduced the number of communication breakdowns, knowing about communication strategies
Kharicha et al. 2013	Seeking medical care (e.g. from general practitioner, optician)	Vision loss	Denial, fear, viewing worsening vision as normal or a part of aging, costs of buying and updating lenses/glasses, mistrust in the commercial motivation of the optical industry	Seeking help depended on significant events and changes (e.g. experiencing difficulty in watching television/driving/reading bus numbers)
Krishnan and Lim 2012	Using subsidized healthcare facilities (e.g. government polyclinics and hospitals)	General health challenges	Low confidence in subsidized medical care providers, language disconcordance, difficulties communicating with doctors led participants to be more likely to be dissatisfied with the quality of healthcare received	Service awareness, financial resources, affordable services
Lau et al. 2014	Seeking help from healthcare professionals	Various (e.g. hypertension, diabetes, arthritis, osteoporosis)	Being married, valuing the idea of keeping personal matters within the family, low self-esteem, impaired body image, fear of intimacy with helping professionals	-
Lawrence et al. 2006	Various (e.g. seeking help from family or general practitioner, religion and self-management)	Depression	Not wanting to rely on family, past experiences of general practitioners not helping, belief that doctors were too busy/overly reliant on medication, limited consultation time, doctors dismissing their compliants, fear of medications creating dependency, concerns about side effects, confusion about the exact role of counsellors, belief one should not share personal problems with strangers, stigma	Being able to share without fear of boring others/becoming a burden/being judged, families providing advice or seeking help on their behalf, seeking friends as source of encouragement/advice/reassurance, access to day centres, not feeling well, belief that doctors could and should help, opportunity to speak to a professional who was distanced from the situation, regular contact with psychiatrists and subsequent experiences of reassurance
Lee et al. 2005	Various (e.g. consulting friends, spouses or health professionals)	Benign prostatic hyperplasia (BPH) and BPH-related symptoms	Attributing symptoms as part of a natural aging process and thus untreatable, feeling embarassed or fearful of cancer	-

Supplemental Table 1. Help-Seeking Characteristics & Factors among Older Adults

Author(s)	Type of help-seeking behavior(s)	Health challenge(s)	Help-seeking Barriers	Help-seeking Facilitators
Lee et al. 2012	Seeing a doctor	Disabilities	Minority status, marital status, lower education, living in Midwest regions/rural areas, older adults with disabilities had significantly higher odds of delaying seeing a doctor due to cost	-
Lee et al. 2020	Accessing healthcare services and financial assistance schemes	Chronic conditions	Low awareness/understanding of services/subsidy schemes/benefits, long wait times, perceived high costs of care, choosing to take personal responsibility over their own health, fear of being a burden, fear of receiving bad news, desire for independence, needs did not always match the services provided, perceived lack of medical expertise by community healthcare providers, low health literacy	Convenience and proximity, perceiving condition to be more serious
Makam et al. 2016	Going to the hospital	Acute myocardial infarction (AMI)	Onset of acute symptoms between 6 PM and 5:59 AM, presence of previously diagnosed multiple comorbidities, hospital presentation with atypical symptoms of AMI, diagnosis of an NSTEMI (non-ST-segment elevation myocardial infarction)	-
Makris et al. 2015	Seeking formal healthcare (e.g. talking to providers, having surgery)	Restricting back pain	Mistrust, negative relationships with providers, placing priority on other comorbidities, beliefs about the inevitability of restricting back pain, providers dismissing/minimizing participants' pain, ageist experiences, negative attitudes toward medication/surgery	Positive relationships with providers
McCabe et al. 2017	Seeking medical attention	Atrial fibrillation (AF)	Inaccurate beliefs/misperceptions about AF, lack of knowledge about AF/symptoms, lack of confidence, unsure of when to seek treatment	Recognition that certain symptoms (e.g. shortness of breath, skipping heartbeats) should be evaluated, availability of family members
McGowan and Midlarsky 2012	Using psychotherapy/counselling	Mental health challenges	Limited openess to mental health interventions, viewing psychotherapists as outsiders, treatment seen as a threat to beliefs, lower stigma tolerance, fear	Perceived need, interpersonal openness, confidence in mental health practitioners
Mechakra-Tahiri et al. 2011	Seeking consultation from a health professional	Depression	Stigma, lack of accessibility, living in rural areas	Perceiving themselves as in poor health, presence of a confidant and emotional/instrumental support *noted differences between men & women
Miller et al. 2016	Receiving informal support from spouses, family members, friends and neighbors	Falls	Wanting to remain independent and self-sufficient, pride, not wanting to owe someone in the future/becoming a burden, feeling devalued	Setting a routine/preset schedule where friends/family were available on specific days each week, mutual dependence/acceptance of help between friends
Mukherjee 2019	Various (e.g. using healthcare services, alternative medical amenities)	General health challenges	Language barriers, lack of accessible information, financial constraints, seeing age and illness as inseparable phenomena, viewing illness as a result of sins, no supports available, not wanting to move out of their home for treatment, cultural beliefs, stigma	Residential proximity of providers, only going to doctors when participants' situation deteriorated

Supplemental Table 1. Help-Seeking Characteristics & Factors among Older Adults

Author(s)	Type of help-seeking behavior(s)	Health challenge(s)	Help-seeking Barriers	Help-seeking Facilitators
Murata et al. 2010	Seeking medical and dental care	Various (e.g. hypertension, visual impairment, arthritis, heart disease, diabetes)	Costs/lower socioeconomic status, distance, lack of transportation, being too busy, conditions not serious enough, living in rural areas	-
Pickard and Guo 2008	Seeking help from a clergy	Mental health challenges	-	Those with less social support/higher-reported levels of stress/reported more frequent religious attendance/engaged in more private religious activities/higher intrinsic religiosity scores more likely to have sought help from clergy
Pickard and Tang 2009	Help-seeking from a clergy compared to other formal sources (e.g. from psychiatrists, self-help groups, or other mental health specialists)	Mental health challenges	-	Religiosity and stress associated with help-seeking from clergy and other formal sources
Polacsek et al. 2019	Seeking formal support (e.g. from professional/private/public health providers, hospitals, community services)	Depression	Stigma, struggling to become self-motivated to seek help, associating aging with mental or physical decline, difficulty accessing formal supports(e.g. far geographical location/long waiting lists/financial cost), perceived low quality of formal support, low awareness of mental health nurses' role and no understanding of how to access them, experiencing ageism, difficulty obtaining an initial diagnosis, lack of informal support	Accepting personal responsibility for help-seeking, improved mental health literacy, access to online information, establishing a therapeutic alliance with a trusted health professional, being actively involved in the decision-making process, optimizing informal support
Schaller et al. 2020	Seeking formal clinical support (e.g. from general practitioners, specialists, or other healthcare personnel)	Sexual problems	Stigma, fear, unsatisfactory approaches by providers, lack of knowledge/competency to request help, sexuality seen as less relevant in relation to other more serious/complex health issues, lack of sexual knowledge as a consequence of cultural/historical factors, experiencing ageism, possible homophobic attitudes/discrimination, healthcare atmosphere not able to provide a caring climate to discuss sensitive/complex matters	Providers initiating the subject of sexuality from a professional standpoint, desire to express personal agency, believing they had a personal duty to ask their doctor, healthcare provider's explicit knowledge and competence (e.g. having a gay doctor)
Schneider et al. 2014	Various (e.g. seeing an audiologist, hearing aid provider, general practitioner, Ear, Nose and Throat specialist, attending a self-help group)	Dual sensory (vision and hearing) impairment	Being able to hear well enough, having more serious priorities to deal with, negative past experiences with hearing aids/service providers, wanting to only deal with vision loss and not hearing, no time	Being participants in the Vision-Hearing Project, hearing loss affecting day-to-day conversations/activities, encouragement from family/friends, hearing getting worse
Stenzelius et al. 2006	Seeking medical help	Urinary symptoms	-	Influence of issues on daily life, storage symptoms and pain symptoms
Stenzelius et al. 2007	Seeking medical help	Fecal incontinence	-	Discomfort and leakage problems

Supplemental Table 1. Help-Seeking Characteristics & Factors among Older Adults

Author(s)	Type of help-seeking behavior(s)	Health challenge(s)	Help-seeking Barriers	Help-seeking Facilitators
Stoller et al. 2011	Consulting a doctor	Various (e.g. chronic conditions, acute symptoms)	Regularly occurring symptoms	Perceiving a change in a symptom, experiencing uncertainty about the efficacy of management strategies, intolerable/intense pain, unknown cause of issues, new/unusual symptoms, persisting/recurring/worsening symptom, intending to use doctor as a gatekeeper (e.g. to receive prescription medications)
Tsai and Tsai 2007	Various (e.g. seeking help from significant others)	General health challenges	Lack of awareness of resources, language barriers, self-perceived health status, having preventive coping strategies, being resigned to one's situation	Convenience of external environmental resources, more human resources, greater understanding of available resources
Waterworth et al. 2018	Contacting a primary nurse by telephone	Multimorbidity (e.g. cardiovascular diseases, diabetes)	Cost, short consultation times, having to make sense of symptoms amidst comorbidities, uncertainty/not knowing who they would approach about concerns or how, attributing symptoms to aging, feeling like nurses could not help as they had been referred to a doctor in the past	Being able to access a nurse by telephone (particularly for those living in rural areas), having a clear understanding of the nurse's role, previous positive experiences of contacting the nurse, awareness that the nurse could be a positive link with the doctor if needed, trust and confidence, feeling heard by the nurses
