# Supplementary material for: Help-Seeking Behaviors Among Older Adults: A Scoping Review
Source: J Appl Gerontol. 2022 Feb 13;41(5):1500–10. doi: 10.1177/07334648211067710 (PMC9024019; doi:10.1177/07334648211067710)
Supplement: sj-pdf-3-jag-10.1177_07334648211067710 - Supplemental Material for Help-Seeking Behaviors Among Older Adults: A Scoping Review [file sj-pdf-3-jag-10.1177_07334648211067710.pdf]

Supplementary File 2. Database Search Queries

MEDLINE/PubMed

1. Search: ("help seek*" OR "treatment seek*" OR "health information seek*" OR "healthcare seek*" OR "care seek*" OR "health seek*") AND ("older adults" OR "older people" OR "elderly" OR "seniors" OR "geriatrics")

("help seek*"[All Fields] OR "treatment seek*"[All Fields] OR "health information seek*"[All Fields] OR "healthcare seek*"[All Fields] OR "care seek*"[All Fields] OR "health seek*"[All Fields]) AND ("older adults"[All Fields] OR "older people"[All Fields] OR "elderly"[All Fields] OR "seniors"[All Fields] OR "geriatrics"[All Fields])

2. Search: ("help seek*" OR "treatment seek*" OR "health information seek*" OR "healthcare seek*" OR "care seek*" OR "health seek*") AND ("older adults" OR "older people" OR "elderly" OR "seniors" OR "geriatrics") AND ("immigrants" OR "ethnic minority" OR "minority populations")

("help seek*"[All Fields] OR "treatment seek*"[All Fields] OR "health information seek*"[All Fields] OR "healthcare seek*"[All Fields] OR "care seek*"[All Fields] OR "health seek*"[All Fields]) AND ("older adults"[All Fields] OR "older people"[All Fields] OR "elderly"[All Fields] OR "seniors"[All Fields] OR "geriatrics"[All Fields]) AND ("immigrants"[All Fields] OR "ethnic minority"[All Fields] OR "minority populations"[All Fields])

Web of Science

1. Search: ("help seek*" OR "treatment seek*" OR "health information seek*" OR "healthcare seek*" OR "care seek*" OR "health seek*") AND ("older adults" OR "older people" OR "elderly" OR "seniors" OR "geriatrics"); 2005-2021

ALL FIELDS: ((“help seek*” OR “treatment seek*” OR “health information seek*” OR “healthcare seek*” OR “care seek*” OR “health seek*”) AND (“older adults” OR “older people” OR “elderly” OR “seniors” OR “geriatrics”))

2. Search: ("help seek*" OR "treatment seek*" OR "health information seek*" OR "healthcare seek*" OR "care seek*" OR "health seek*") AND ("older adults" OR "older people" OR "elderly" OR "seniors" OR "geriatrics") AND ("immigrants" OR "ethnic minority" OR "minority populations"); 2005-2021

ALL FIELDS: (("help seek*" OR "treatment seek*" OR "health information seek*" OR "healthcare seek*" OR "care seek*" OR "health seek*") AND ("older adults" OR "older people" OR "elderly" OR "seniors" OR "geriatrics") AND ("immigrants" OR "ethnic minority" OR "minority populations"))

PsycInfo

1. Search: ("help seek*" OR "treatment seek*" OR "health information seek*" OR "healthcare seek*" OR "care seek*" OR "health seek*") AND ("older adults" OR "older people" OR "elderly" OR "seniors" OR "geriatrics"); 2005-2021

((“help seek*” OR “treatment seek*” OR “health information seek*” OR “healthcare seek*” OR “care seek*” OR “health seek*”)) AND ((“older adults” OR “older people” OR “elderly” OR “seniors” OR “geriatrics”))

2. Search: ("help seek*" OR "treatment seek*" OR "health information seek*" OR "healthcare seek*" OR "care seek*" OR "health seek*") AND ("older adults" OR "older people" OR "elderly" OR "seniors" OR "geriatrics") AND ("immigrants" OR "ethnic minority" OR "minority populations"); 2005-2021

((“help seek*” OR “treatment seek*” OR “health information seek*” OR “healthcare seek*” OR “care seek*” OR “health seek*”)) AND ((“older adults” OR “older people” OR “elderly” OR “seniors” OR “geriatrics”)) AND ((“immigrants” OR “ethnic minority” OR “minority populations”))

CINAHL

1. Search: ("help seek*" OR "treatment seek*" OR "health information seek*" OR "healthcare seek*" OR "care seek*" OR "health seek*") AND ("older adults" OR "older people" OR "elderly" OR "seniors" OR "geriatrics"); 2005-2021

((“help seek*” OR “treatment seek*” OR “health information seek*” OR “healthcare seek*” OR “care seek*” OR “health seek*”)) AND ((“older adults” OR “older people” OR “elderly” OR “seniors” OR “geriatrics”))

2. Search: ("help seek*" OR "treatment seek*" OR "health information seek*" OR "healthcare seek*" OR "care seek*" OR "health seek*") AND ("older adults" OR "older people" OR "elderly" OR "seniors" OR "geriatrics") AND ("immigrants" OR "ethnic minority" OR "minority populations"); 2005-2021

((“help seek*” OR “treatment seek*” OR “health information seek*” OR “healthcare seek*” OR “care seek*” OR “health seek*”)) AND ((“older adults” OR “older people” OR “elderly” OR “seniors” OR “geriatrics”)) AND ((“immigrants” OR “ethnic minority” OR “minority populations”))

Scopus

1. Search: ("help seek*" OR "treatment seek*" OR "health information seek*" OR "healthcare seek*" OR "care seek*" OR "health seek*") AND ("older adults" OR "older people" OR "elderly" OR "seniors" OR "geriatrics")

TITLE-ABS-KEY ("help seek*" OR "treatment seek*" OR "health information seek*" OR "healthcare seek*" OR "care seek*" OR "health seek*") AND TITLE-ABS-KEY ("older adults" OR "older people" OR "elderly" OR "seniors" OR "geriatrics")

2. Search: ("help seek*" OR "treatment seek*" OR "health information seek*" OR "healthcare seek*" OR "care seek*" OR "health seek*") AND ("older adults" OR "older people" OR "elderly" OR "seniors" OR "geriatrics") AND ("immigrants" OR "ethnic minority" OR "minority populations")

TITLE-ABS-KEY ("help seek*" OR "treatment seek*" OR "health information seek*" OR "healthcare seek*" OR "care seek*" OR "health seek*") AND TITLE-ABS-KEY ("older adults" OR "older people" OR "elderly" OR "seniors" OR "geriatrics") AND TITLE-ABS-KEY ("immigrants" OR "ethnic minority" OR "minority populations")
